# Computational Investigation on the Biomechanical Responses of the Osteocytes to the Compressive Stimulus: A Poroelastic Model

**DOI:** 10.1155/2018/4071356

**Published:** 2018-01-18

**Authors:** Liping Wang, Jianghui Dong, Cory J. Xian

**Affiliations:** ^1^Sansom Institute for Health Research, School of Pharmacy and Medical Sciences, University of South Australia, Adelaide, SA 5001, Australia; ^2^School of Natural and Built Environments, University of South Australia, Adelaide, SA 5095, Australia

## Abstract

Osteocytes, the major type of bone cells embedded in the bone matrix and surrounded by the lacunar and canalicular system, can serve as biomechanosensors and biomechanotranducers of the bone. Theoretical analytical methods have been employed to investigate the biomechanical responses of osteocytes in vivo; the poroelastic properties have not been taken into consideration in the three-dimensional (3D) finite element model. In this study, a 3D poroelastic idealized finite element model was developed and was used to predict biomechanical behaviours (maximal principal strain, pore pressure, and fluid velocity) of the osteocyte-lacunar-canalicular system under 150-, 1000-, 3000-, and 5000-microstrain compressive loads, respectively, representing disuse, physiological, overuse, and pathological overload loading stimuli. The highest local strain, pore pressure, and fluid velocity were found to be highest at the proximal region of cell processes. These data suggest that the strain, pore pressure, and fluid velocity of the osteocyte-lacunar-canalicular system increase with the global loading and that the poroelastic material property affects the biomechanical responses to the compressive stimulus. This new model can be used to predict the mechanobiological behaviours of osteocytes under the four different compressive loadings and may provide an insight into the mechanisms of mechanosensation and mechanotransduction of the bone.

## 1. Introduction

Bone is a dynamic biological structure that can adapt to its mechanical environment by changing its structure [1, 2]. The mechanical stimulation has now been recognized vital for regulating bone remodelling processes of bone formation (by bone-forming cells osteoblasts) and resorption (by bone-resorptive cells osteoclasts) and thus bone microstructure, bone mass, and bone strength [3]. Osteocytes are seen as the main candidates for mechanosensory effects of the bone, which are the most numerous bone cells (making up about 90–95% [4, 5] of all bone cells, with osteoblasts and osteoclasts together only making up a total of up to 6% [6]).

Osteocytes are known to regulate bone remodelling and are located within their lacunae surrounded by the perilacunar matrix (PCM) embedded in the bone matrix (extracellular matrix or ECM). Osteocytes are connected to each other with slender cell processes located within small tubes called the canaliculi within the bone matrix. It is believed that, through this osteocyte cell body-cell process-lacunar-canalicular system, the osteocytes are the mechanosensors/mechanotranducers in bones, which can sense the mechanical loadings and transduce them into biochemical signals regulating bone remodelling [7–11].

To investigate bone tissue mechanosensing/mechanotransduction and biomechanical behaviours of osteocytes, a number of researchers have studied the osteocyte-lacunar-canalicular system by theoretical analytical models. The mathematical model has been a good tool to explain the phenomenon of mechanotransduction in the lacunar-canalicular system [12], which is well accepted to be induced by flow movement-like fluid shear stresses [13–19]. Combined with microfocus computer tomography (mCT), the finite element (FE) modeling can be a biomechanical analysis tool to study bone [20]. In addition, the FE method can be used to simulate blood flow, regeneration processes, bone remodeling process, and heat transfer and evaluate bone strength [21, 22]. McCreadie and Hollister (1997) were the first researchers to study the mechanical behaviours of lacunae and osteocytes by using an idealized 3D linearly isotropic material FE model [23]. Bonivtch et al. (2007) analysed the microstructural responses of the osteocyte lacuna by using a parametric linearly isotropic material FE model [24]. Sanz-Herrera et al. (2008) developed the FE and the Voxel-FE models at the macroscopic and microscopic scales [25]. More recently, with the development of computer and FE analysis software, the FE analysis has been widely applied to investigate the biomechanical behaviours of osteocytes [26–29]. 3D linearly isotropic material FE models of osteocytes have been developed including an idealized model and biorealistic (confocal image-derived) model [26]. A linearly isotropic material 3D biorealistic osteocyte FE model was created based on synchrotron X-ray phase nanotomography [30]. Strain amplification analysis was conducted on an osteocyte under static and cyclic loading using an idealized linearly isotropic material 3D FE model [28]. Based on quasi-3-dimensional (quasi-3D) cell microscopy, a fluid–structure interaction FE method was used to study viscoelastic property [29]. Thus, the FE model analysis is an effective alternative method for the in vitro biomechanical studies [22].

Since nutrients and exchanges of metabolic products or biochemical signals of osteocytes depend on the movement of interstitial fluid [31], basically, nutrients are transported by both fluid flow and diffusion within the bone [32–34]. Reich et al. (1990) [35] and Reich and Frangos (1991) [36] were the first to conduct fluid flow studies on bone cells in culture, and they found that osteoblasts and endothelial cells had similar responses to fluid shear stress excitation. Small molecules (e.g., amino acids) can be diffusively transported alone to the osteocytes [37]. The solute transport occurred through the lacunar-canalicular system under cyclic loading, when the concentrations were different in the lacunar flow between the inward and outward [33]. Goulet et al. [32] used a homogeneous model and a vascularized model to demonstrate the bulk fluid movement and fluid exchange between the canals and the lacunocanalicular porosity. The process of the diffusional mixing was very fast and the numerous osteocyte lacunae are used as mixing chambers [17]. Osteocytes were proposed to be stimulated by relatively small fluid shear stresses acting on the membranes of the osteocytes [14]. In addition, since the passage of interstitial pore fluid adjacent to dendritic cell structure occurs in the porosity of the bone hierarchical microstructure, interaction of fluid and the osteocyte-lacunar-canalicular system has been regarded to play an important role in bone tissue mechanosensing and mechanotransduction. Thus, it has been recognized that understanding the actual physics of flows in bone is important for the analyses of bone remodelling and the bone hierarchical microstructure [38].

Poroelasticity is a well-developed theory for investigating the interaction of fluid and solid phases in a fluid-saturated porous medium [39–41], which has been an important means applied in research into bone and the osteocyte-lacunar-canalicular system [31, 42–45]. Based on the theory of poroelasticity, the poroelastic properties include drained shear modulus, drained and undrained Poisson's and Skempton's coefficient, and permeability coefficient [39, 46, 47]. This poroelastic theory was the first model used to study the small-scale fluid mechanics within the lacunar-canalicular porosity using Brinkman's equation (Darcy's law and Stokes equation). The poroelastic properties should be taken into account in the study of the osteocyte-lacunar-canalicular system [48, 49]. A computational model was developed to explore load-induced fluid flow in bone as a mechanotransduction mechanism under physiologically realistic loading with different frequencies [50]. Subsequently, a 2D anisotropic poroelastic model was used to study the local fluid flow characteristics in the vicinity of a single lacuna [19]. More recently, Nguyen et al. studied the mechanical behaviours of one single chondrocyte by using poroviscohyperelastic model [45].

However, the material properties of the models in some of the previous studies [23, 24, 26, 51] were assumed solid, in which the fluid or the interaction between fluid and solid matrix was ignored. Although the poroelastic properties were considered in some studies, these models were theoretical models [42, 47, 52] or 2D FE models [19]. In addition, while foregoing FE models had focused on the physiological compressive loading, there has been little attempt reported investigating/comparing the effects of disuse (150 microstrains), physiological (1000 microstrains), overuse (3000 microstrains), and pathological overload (5000 microstrains) compressive loads [53, 54] on the osteocyte-lacunar-canalicular system using FE model analyses. In the current study, an idealized 3D poroelastic FE model was developed to investigate the biomechanical responses of the osteocyte-lacunar-canalicular system when subjected to four different compressive loads. Since it has been stated that, in general, the disuse load was under 200 *με*, the physiological load was between 200 and 2500 *με*, and the overuse loading range was 2500–5000 *με* [53], consistently, the osteocyte-lacunar-canalicular system FE model in the current study was selected to be under the global compressive loads of 150 microstrains (disuse), 1000 microstrains (physiological), 3000 microstrains (overuse), and 5000 microstrains (pathological overload), respectively. Under these loads, the following three analyses were made: (1) the distributions of the maximum principal strains; (2) the distributions of the maximum pore pressures; and (3) the maximum fluid velocities of the FE model.

## 2. Materials and Methods

### 2.1. Geometry and a 3D Model of the Idealized Osteocyte-Lacunar-Canalicular System

**Figure 1:**
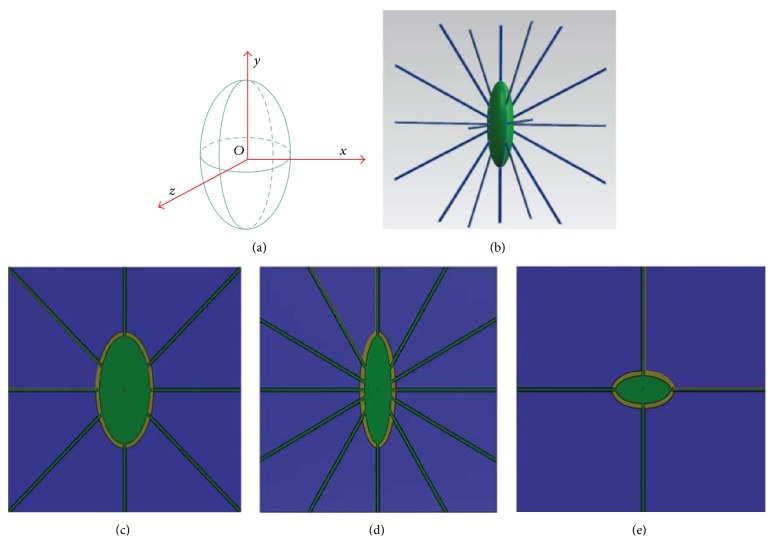
3-dimensional schematic representations of an idealized osteocyte-lacunar-canalicular system. (a) A schematic diagram of an osteocyte lacuna, where *z* is the intermediate axis, *y* the major axis, and *x* the minor axis of the lacuna in the local coordinate system. Schematic diagrams of osteocyte lacuna and canaliculi, showing (b) osteocyte cell body (green) with processes (blue); (c) in *x*-*y* plane; (d) in *z*-*y* plane; and (e) in *x*-*z* plane.

**Figure 2:**
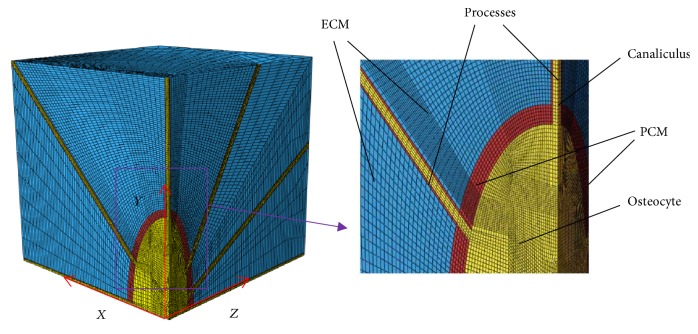
One-eighth symmetry model applied in the 3-dimensional finite element meshing analyses of the idealized osteocyte-lacunar-canalicular system, showing the geometric locations of extracellular matrix (ECM), perilacunar matrix (PCM), osteocyte cell body, canaliculi, and cell processes.

The osteocyte-lacunar-canalicular system was idealized as a 3D structure (Figure 1). The osteocyte lacuna was described as a triaxial ellipsoid, which can be expressed as follows [55].

(1) $$\begin{array}{c} {\left(\;\frac{x}{l}\;\right)}^{2}+{\left(\;\frac{y}{m}\;\right)}^{2}+{\left(\;\frac{z}{n}\;\right)}^{2}=1, \end{array}$$

where *l*, *m*, and *n* are the semiaxes of the osteocyte lacuna in *x*, *y*, and *z* directions, respectively. *x*, *y*, and *z* are the minor, major, and intermediate axes of the osteocyte lacuna in the local coordinate system, respectively (Figure 1(a)). The idealized model is comprised of a number of parts, that is, ECM, PCM, osteocyte cell body, canaliculi, and cell processes [28] (Figures 1 and 2 and Table 1).

The side length of each direction of the ECM cube is 43 *μ*m for human [55] and is the outermost layer of the system, which was modeled as an external solid layer, consisting of 77480 eight-node solid brick elements (Figure 2 and Table 1).

**Figure 3:**
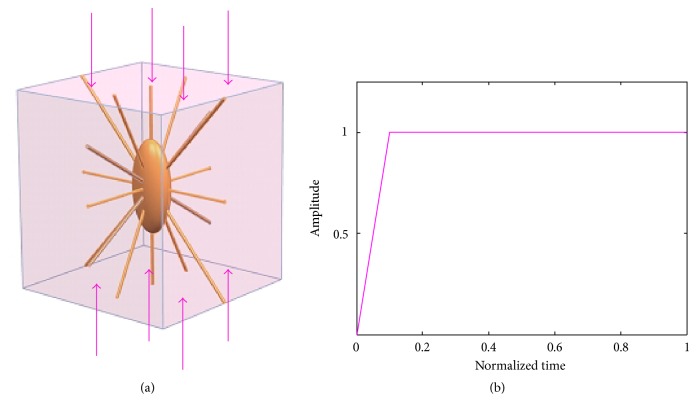
Conditions of compressive loading for the osteocyte-lacunar-canalicular system finite element model. (a) Uniaxial compressive loading; (b) loading mode.

The middle layer of the system is the PCM with canaliculi that separates the ECM and the osteocyte cell body [2] (Figure 3). Here, it was modeled by two parts, namely, the PCM, ~0.5–1 *μ*m thick layer [56], and the canaliculi, straight cylindrical channels with a diameter of 0.25 *μ*m [1, 57]. Here, both the PCM and canaliculi are solid and consist of 7183 eight-node brick elements in total (Table 1).

In this study, the simulations were conducted using the software ABAQUS 6.12 (SIMULIA, Providence, RI, USA) to assume fully saturated media. In the simulations, only 1/8 symmetry model was applied in the whole FE analyses because the model is symmetrical (Figure 2). Also, the elements are eight-node C3D8R elements.

### 2.2. Properties of Poroelastic Materials Applied in the Osteocyte-Lacunar-Canalicular System

The material properties (isotropic material) used to represent the osteocyte-lacunar-canalicular system are listed in Table 2. In this study, to develop the model, ECM, PCM, and osteocyte cell body were assumed to be poroelastic materials. In the poroelastic constitutive law, the total stress (*σ*_*T*_) acting at a point results from the combination of an effective stress (*σ*_*E*_) and a pore pressure (*p*), respectively, carried out by the solid matrix and the fluid phase. Their relationship is shown in (2). In addition, the poroelastic formulation is based on the field variables *u* (solid displacement) and *p* (pore pressure) in ABAQUS (3).

(2) σT=σE−pI

(3) ∇u+∇q→=0,

where *I* is the unit tensor, ∇ represents gradient, and $\vec{q}$ is fluid mass flow.

The fluid mass flow $\vec{q}$ is relative to the pore pressure and can be computed according to Darcy's generalised law for flow through porous media, and the calculated formula can be expressed as follows.

(4) $$\begin{array}{c} \vec{q}=-k\nabla p. \end{array}$$

The fluid flow can also be expressed as follows:

(5) $$\begin{array}{c} \vec{q}=\vec{v}\times n_{f}, \end{array}$$

where *k* is the permeability (which is assumed isotropic in this study), ∇*p* is the gradient of pore pressure, $\vec{v}$ is fluid velocity, and *n*_*f*_ is fluid volume fraction (porosity).

Material parameters involved in the poroelastic formulation are Young's modulus (*E*), Poisson ratio (*υ*), porosity (*n*_*f*_) [which is related to the void ratio (*e*) and can be expressed as (6) and (7)], and permeability (*k*, unit of m^2^). The strain-dependent permeability with the exponential constitutive law has been developed [64–66]. The permeability *k* is assumed as the function of void ratio and may be expressed as (8) [67]. This function can be implemented in ABAQUS software package.

(6) nf=VfluidVfluid+Vsolid=VfluidVtotal=e1+e

(7) e=VfluidVsolid=VfluidVtotal−Vsolid=nf1−nf

(8) k=k0e1+e0e01+e2exp⁡M1+e1+e0−1,

where *V*_fluid_ is the volume of void-space (such as fluids), *V*_solid_ is the volume of solids, *V*_total_ is the total or bulk volume, *e* is void ratio, *k*_0_ is the initial permeability, *e*_0_ is the initial void ratio, and *M* is a constant and needs to be determined [68].

### 2.3. Boundary and Loading Conditions

In order to investigate the biomechanical behaviours of the osteocyte-lacunar-canalicular system, the compressive loadings were applied to the 3D FE model. In this study, the displacements were used as loadings rather than the forces. In the current study, the uniaxial ramped static loading was applied on the model.

Uniaxial compressive loads were applied in the long bone axis (Figure 3), with global displacement loads of compressive loads applied, respectively, at 150 microstrains (disuse), 1000 microstrains (physiological), 3000 microstrains (overuse), and 5000 microstrains (pathological overload) [53, 62]. In order to prevent any relative movements in all simulations and to ensure the PCM attaching to the osteocyte and ECM, they were assumed being bonded perfectly. Thus, the displacement should be the same for the interface surfaces. ECM, PCM and canaliculi, and osteocyte cell body and processes were assumed being bonded perfectly. Distributions of the maximum principal strains of the osteocyte-lacunar-canalicular system FE model were simulated under these different loads.

To prevent rigid body motion, the symmetrical boundary conditions were applied to the nodes on the opposing faces of the applied displacement loading, and only 1/8 of the symmetry model was used in the FE analyses. Furthermore, the initial fluid pore pressure was assumed to be zero and imposed on the external surfaces of the model due to the lack of osmotic pressure in the osteocyte-lacunar-canalicular system [59].

## 3. Results

### 3.1. Validations of the Osteocyte-Lacunar-Canalicular System FE Model

**Figure 4:**
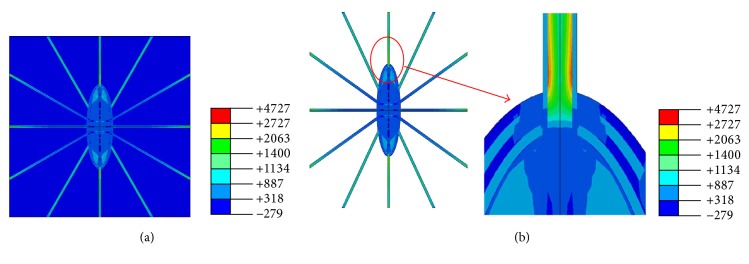
Strain distributions of finite element model under 1000-microstrain global loading. (a) Perilacunar matrix and osteocyte; (b) extracellular matrix.

Distributions of the maximum principal strains of the osteocyte-lacunar-canalicular system FE model were predicted under global compressive loads of 150 microstrains (disuse), 1000 microstrains (physiological), 3000 microstrains (overuse), and 5000 microstrains (pathological overload), respectively. The maximum principal strains of the FE model were found to be ~633, 4272, 12820, and 21528 microstrains, respectively. The strain distribution of the model under the 1000-microstrain compressive loading is shown in Figure 4. The peak amplitudes of the cellular processes were found higher than those of the ECM, PCM, and the cell body (Figure 4). Moreover, the strain amplification factor of simulation was ~4.3 (Table 3). These findings are in agreement with those of the previous studies [2, 24, 63, 69], where the maximum strain amplification factors were measured to be ~7.5 in the in vitro measurement. While 7.5 was the maximum value of one of the many tested osteocytes, the average strain amplification factor was ~4 [63, 69].

**Figure 5:**
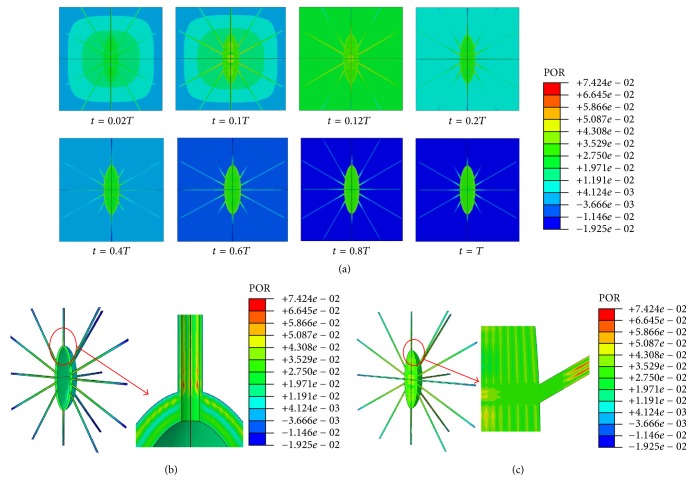
Pore pressure distributions of the osteocyte-lacunar-canalicular system under 1000-microstrain compressive loading. (a) The maximum pore pressure when *t* = 0.02, 0.1, 0.12, 0.2, 0.4, 0.6, 0.8, or 1.0*T* (total period of loading); (b) pore pressure distributions at canaliculi and perilacunar matrix when *t* = 0.1*T*; (c) pore pressure distributions in osteocyte and processes when *t* = 0.1*T*.

The maximum pore pressures on the cell and processes were 8.98, 74.24, 175.9, and 285.7 kPa when the model was subjected to global compressive loads of 150, 1000, 3000, and 5000 microstrains, respectively.  Figure 5 shows the pore pressure and distribution results of the FE model when under 1000-microstrain compression loading. As shown, the predicted maximum pore pressure of the model occurs at 0.1 of the total period of loading (0.1*T*), and the maximum pore pressure is located at the junction areas of canaliculi, PCM, and processes.

Previously, the maximum pore pressure of the cortical bone was found to be up to 250 kPa under 1 MPa uniaxial stress [50], and the maximum hydraulic pressure of the lacunar-canalicular system was up to 5 MPa when an osteon was subjected to 1 Hz 1000 microstrain compression [15]. Thus, the predicted pore pressure values of our model (i.e., 8.98, 74.24, 175.9, and 285.7 kPa for the compressive loads of 150, 1000, 3000, and 5000 microstrains, respectively) are within the range of predicted values of these previous studies.

**Figure 6:**
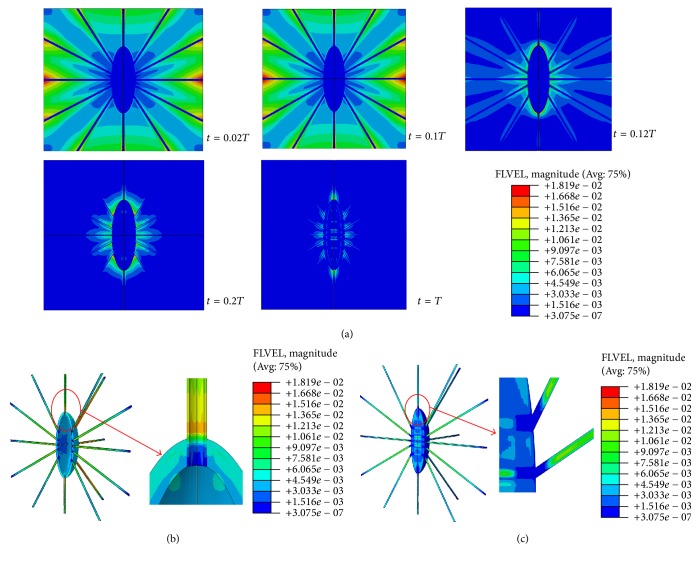
Fluid velocity distributions of the osteocyte-lacunar-canalicular system under the 1000-microstrain compressive loading. (a) Fluid velocities when *t* = 0.02, 0.1, 0.12, 0.2, and 1.0*T*, where *T* is the relative period of loading; (b) fluid velocity distribution at canaliculi and perilacunar matrix when *t* = 0.1*T*; and (c) fluid velocity distribution at the osteocyte and processes.

The maximum fluid velocities of the FE model were found to be 2.69, 18.19, 56.65, and 97.98 *μ*m/s when the model was subjected to global compressive loads of 150, 1000, 3000, and 5000 microstrains, respectively.  Figure 6 displays the fluid velocities of the FE model under the 1000-microstrain compression load. The fluid velocities changed only slightly after *t* = 0.2*T*. In a previous study, the peak fluid flow velocity in canaliculi of the bone lacunar-canalicular system was ~60 *μ*m/s under a surface strain of 400-microstrain loading [70]. Recently, Wang et al. presented the range of fluid flow velocity of 13.1–69.3 *μ*m/s under 298- and 510-microstrain loading [71]. Compared to these experimental and theoretical velocity values, our predicted maximum fluid velocities of the FE model are within the reasonable and comparable ranges.

### 3.2. Distributions of the Maximum Principal Strains, Maximum Pore Pressures, and Maximum Fluid Velocities

**Figure 7:**
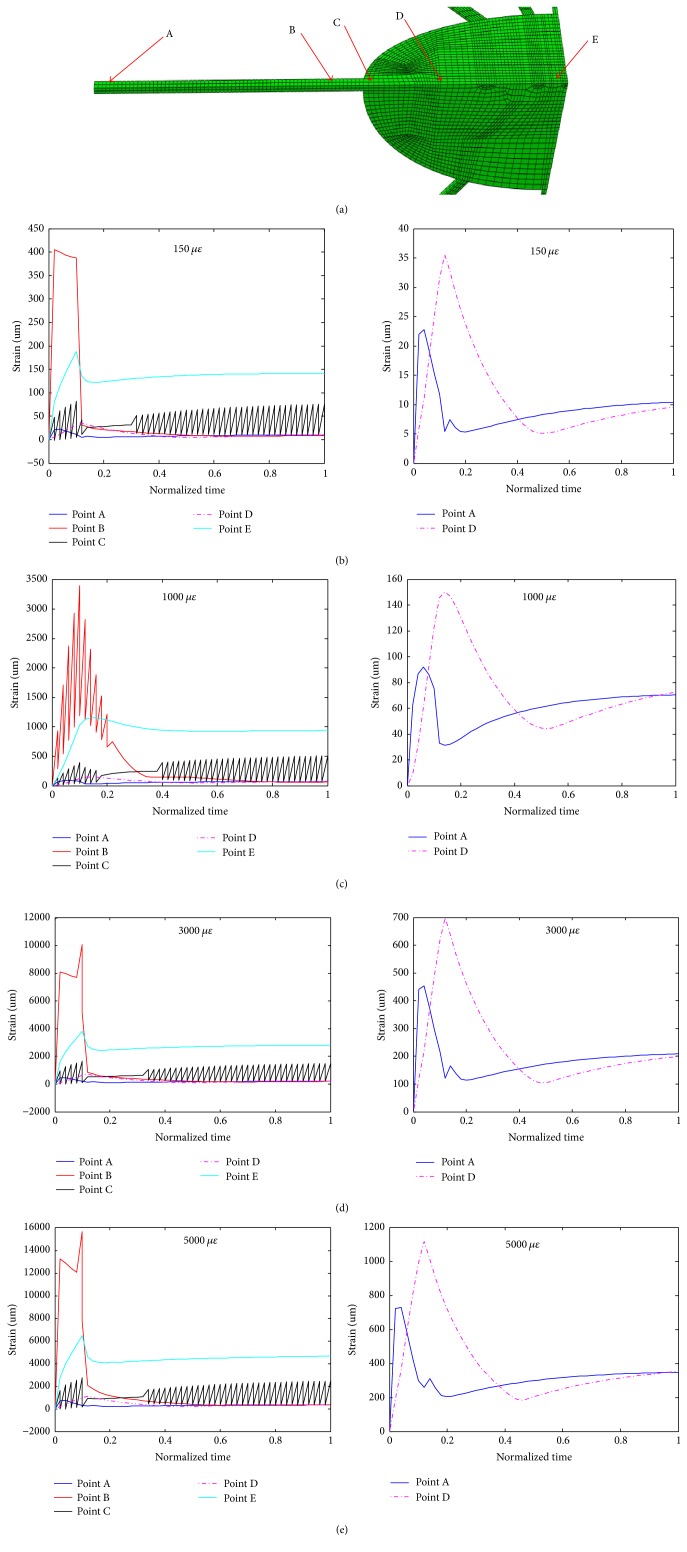
Detailed analyses of the maximum principal strains versus loading time at different locations (a) of the osteocyte cell body and a process when under different compressive loads ((b), (c), (d), and (e) for 150, 1000, 3000, and 5000 microstrains, respectively).

In order to investigate the biomechanical responses of the cell body and processes, five points of the model in one straight line were selected for analyses (Figure 7(a)). Because the geometry of the osteocyte-lacunar-canalicular model is symmetrical, only one process and 1/8 the cell body were analysed. Point A is located at the end of the process which is near to the loading surface; point B is near to the junction of the process and PCM; point C is at the top of the cell body and near the junction of the process and PCM; point D is about the middle of 1/8 cell body; and point E is at the bottom of 1/8 cell body (Figure 7(a)).

Detailed analyses of the maximum principal strains with loading time at these five different locations are shown in Figures 7(b)–7(e) when the model was subjected to different compressive loads. As shown, the strain value of point E is bigger than those of the other points in the steady state; point B has the oscillation in the early phase and point C has the oscillation in most phases of loading time; and the values of points A and D are smaller than those of other points. In Figure 7, some oscillations occurred at point C, which is at the top of the cell body and near the junction of the cell process and perilacunar matrix (PCM). Being at the junction of the cell body, process, and PCM, whose material properties were different, the responding displacement/strain of point C may thus oscillate under mechanical loads.

**Figure 8:**
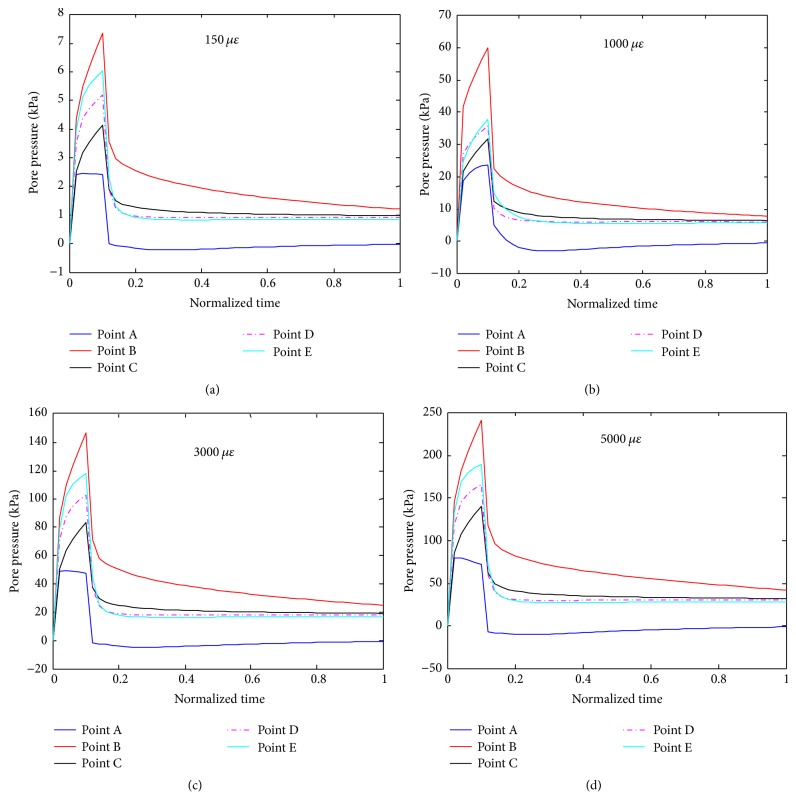
Detailed analyses of pore pressures versus loading time at different locations (defined in Figure 7(a)) of the osteocyte cell body and a process when the system was under different compressive loads ((a), (b), (c), and (d) for 150, 1000, 3000, and 5000 microstrains, respectively).

Figure 8 shows results of detailed analyses of the pore pressure distributions of the cell body and a process at the five different locations (points A, B, C, D, and E) when the FE model was under the four different compressive loadings. As shown, the pore pressure of point B is larger than those of the other points and that at point A it is smallest among these points.

**Figure 9:**
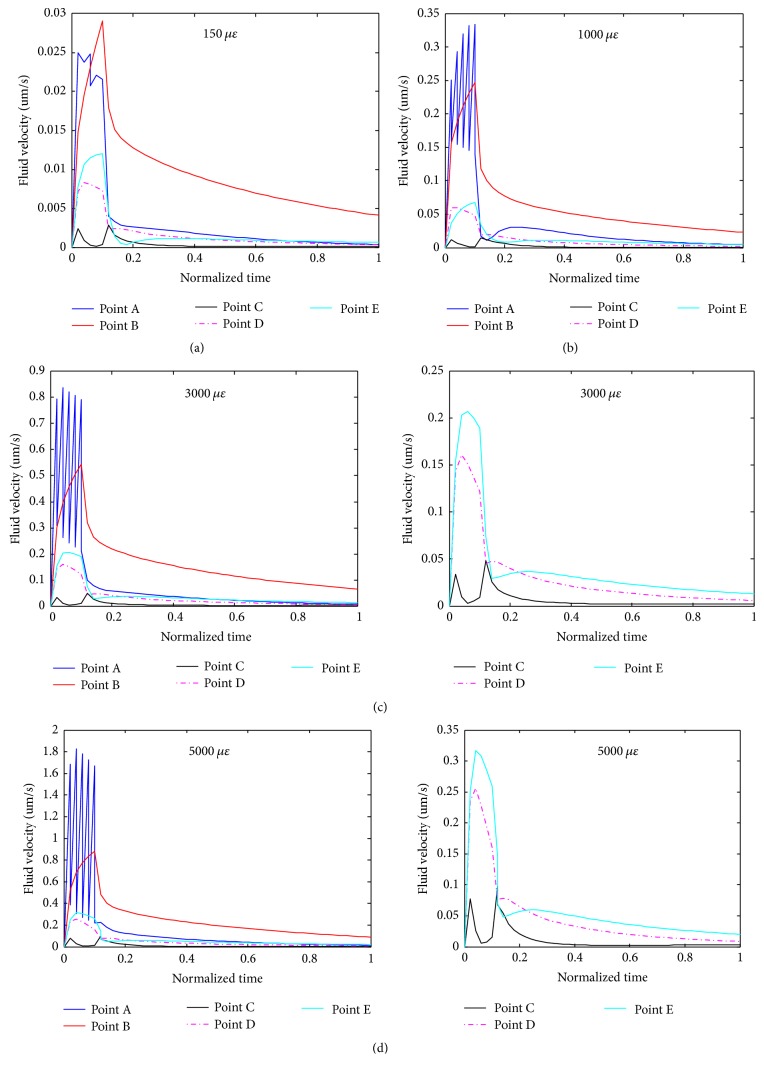
Detailed analyses of fluid velocities versus loading time at different locations (defined in Figure 7(a)) of the osteocyte cell body and a process when the system was under different compressive loads ((a), (b), (c), and (d) for 150, 1000, 3000, and 5000 microstrains, respectively).

Figure 9 presents results of detailed analyses of the fluid velocities of different locations (points A, B, C, D, and E) of cell body and a process when the model was under different compressive loadings. Fluid velocity of point B is larger than those of the other points in the steady state. In the early response phase, the fluid velocity of point A appears in oscillation and then reaches a value in the steady state. Point A is in distal end of the process. The fluid velocity of point A is more sensitive than other points during the ramp increase.

## 4. Discussion

It is now widely believed that fluid that flows through the osteocyte-lacunar-canalicular system can affect and control the bone adaptation since when the osteocytes are stimulated by fluid stress, they can produce biological signals that guide the recruitment/activity of osteoclasts and/or osteoblasts on the surface of the bone [47]. Thus, the fluid embodied in osteocyte-lacunar-canalicular system is vital for the system to regulate bone remodelling [72]. Previously, various models including the FE analysis model have been used to study biomechanical behaviours of the osteocyte-lacunar-canalicular system. However, these previous studies have not taken the poroelastic properties into the FE model analysis. The current study has developed a FE model with one triaxial lacunar osteocyte ellipsoid and, by using a 3D poroelastic model under four different compressive loads, investigated the biomechanical responses (strain, pore pressure, and fluid velocity) of the osteocyte-lacunar-canalicular system. It is now known that different biomechanical responses can be produced by different mechanical stimulations [3], including the tensile loading when the bone is bended or pulled away from itself and the compressive loading caused by the force pushing the bone together (e.g., the animal's weight in the axial load component) [73]. Among these, the compressive load has been widely studied in osteocytes by using the FE analysis method [24, 26–28]. Although several analyses of the strain amplification in osteocytes using the poroelastic models have been reported [18, 74, 75], these studies had focused on theoretical models, and the relative contribution of strain amplification has not been reported with 3D poroelastic FE models that are subjected to mechanical loading-like strains. In the current study, four different degrees of compressive strain loads were applied to our 3D poroelastic FE model to investigate the biomechanical responses of the osteocyte-lacunar-canalicular system, that is, under 150 microstrains (disuse), 1000 microstrains (physiological), 3000 microstrains (overuse), and 5000 microstrains (pathological overload), respectively.

The simulation results of our poroelastic FE model under the compressive loads were found to be in good agreement with the previous results [24, 26, 30, 63, 69, 76]. For example, our predicted maximum principal strain was ~12820 microstrains under 3000-microstrain compressive load, and the corresponding result from Verbruggen et al. (2012) was ~6600–12600 microstrains [26]. In addition, Bonivtch et al. (2007) predicted the local strain was ~4000–6000 microstrains for the osteocyte lacuna under a 2000-microstrain compressive load [24]. Thus the predicted results from our poroelastic FE model are consistent with data from the literature. Since there is no validation data for the idealized model in the existing literature, our predicted result for the 5000-microstrain compressive load cannot be compared. A study indicated that the maximum principal strain was 5-fold higher than the global strain load [76]. In a recent biorealistic osteocyte model study, the local strain amplification factors were up to 10 and up to 70 in the ECM and in the osteocytes, respectively, and the peak amplitude of this model was 50,000–70,000 microstrains under the 1000-microstrain global compression [30]. Data from the current study indicates that the strain amplification factors only changed very slightly with the different global loads applied, suggesting the strain amplification factor is not sensitive to the global loads.

Pore fluid pressure occurred when the osteocyte-lacunar-canalicular system is subjected to loads [42]. The osteocyte-lacunar-canalicular system is a high-fluid-pressure domain because the pore size of the system is very small, leading to a slow decay of a pressure pulse [31]. The current study has computed the pore fluid pressure distributions in the osteocyte cell body and processes under the compressive loadings applied. The maximum pore fluid pressures of cell body and processes were found to be 8.98, 74.24, 175.9, and 285.7 kPa when under 150, 1000, 3000, and 5000 microstrains, respectively. Previously, a modest pore fluid pressure in the lacuna was found to be ~9.3 kPa when under a 100-microstrain compressive load [19], and the maximal pore fluid pressure magnitude was ~0.86, 8.58, and 85.35 kPa when the permeability was 10^−18^, 10^−19^, and 10^−20^ m^2^, respectively [42]. In addition, maximum pore pressure was up to 250 kPa when the cortical bone was subjected to 1 Mpa uniaxial stress [50], and the maximum hydraulic pressure of lacunar-canalicular system was up to 5 MPa when an osteon was under 1000-microstrain compression at 1 Hz frequency [15]. Our predicted results of pore fluid pressure are thus consistent with the findings of these previous studies. In addition, these data suggest that the pore pressure increases with the increasing global loading.

Furthermore, using the present model, fluid velocity was also predicted and simulation was conducted for fluid flow within the osteocyte-lacunar-canalicular system in situ. In our model, the maximum fluid velocities were 2.69, 18.19, 56.65, and 97.98 *μ*m/s under 150, 1000, 3000, and 5000 microstrains, respectively, suggesting that the fluid velocity increases with the increasing global loading. Fluid velocity is another important characteristic of the response of an osteocyte to the mechanical loading stimuli, since fluid movement within the lacunar-canalicular system caused by mechanical loading can cause small deformations of bone, deliver nutrients to, and remove wastes from the osteocytes [77], and the small fluid shear stress acting on PCM and the osteocyte processes can regulate the lacunar-canalicular system [14].

Moreover, the distributions of the strain, pore pressure, and fluid velocity of five selected points in the cell and a process were investigated over the whole loading time period and under different degrees of compressive loading. It was found that the values of these parameters increased firstly and reached the peak values at about the 0.1*T*, followed by declining and finally reaching the steady states. Overall, these trends of changes are coincident with the mode of loading with the load increasing starting from the initial point and reaching the highest load at 0.1*T*. Furthermore, among the selected locations analysed, the proximal side (near to the cell) of a process was found to bear the highest local strain, pore pressure, and fluid velocity. In addition, among the various degrees of loads applied, it was found that the degrees of biomechanical responses increase with mechanical loads. In other words, the local strain, pore pressure, and fluid velocity of the cell and a process increase with the global loading. Because the loading time and the ramp time were kept the same, the high compressive loading occurred with sharper loading ramps and caused much higher velocity, pressure, and principal strains peaks.

In the current study, the poroelastic material property was applied in all simulations. The osteocyte-lacunar-canalicular system is considered to be fully saturated, having only a solid matrix phase and a fluid phase with no air voids, and it consists of the fluid spaces surrounding the osteocytes and their processes. In most previous studies, the porosity of osteocyte-lacunar-canalicular system was assumed to be 0.05 [39, 47, 72, 78]. However, in some studies, the values were variable, being as low as 0.023 in man [79], 0.042 in dogs [80], and even 0.007 in mice [81], and as high as 0.14 in rats [82].

Moreover, the permeability (the ease with which the fluid can move within a porous system) which is related to porosity, is also a very important measure for the poroelastic model. In the current study, viscosity was assumed 0.001 Pa s (i.e., viscosity of water). Permeability depends on the number, orientation, and size of the canaliculi, as well as on the amount of filling by osteocytes and their processes. While it is too hard to determine it directly, there have been estimations of permeability of the osteocyte-lacunar-canalicular systems ranging over 8 orders of magnitude [52], with the estimated values ranging from 5 × 10^−25^ to 7.172 × 10^−17^ (m^2^) [19, 42, 55, 72, 78, 83, 84]. The value of permeability was determined or estimated by different methods, for example, the theoretical method [19, 52, 83], experimental method [47, 78], and nanoindentation technology [85]. Such variation in the reported/estimated values may be a consequence of many factors, including differences in theoretical assumptions/boundary conditions, experimental errors associated with the nested porosities in bone which are difficult to isolate, the freshness of the tissue tested, and the presence of the soft tissues inside the lacunae and canaliculi [31]. According to the analysis from a previous study [31], to be able to produce fluid flow inside canaliculi and stimulate osteocytes, the permeability in the lacunar-canalicular domain should be ~10^-20 ^m^2^ or smaller. In our study, the permeability values are 0.6 × 10^−19^, 4 × 10^−20^, and 5.0 × 10^-15 ^m^2^ in the cell body and process, in PCM and canaliculi, and in the ECM, respectively. The permeability is not a constant value and thus the strain-dependent permeability was implemented in the present FE model. Our result is consistent with Darcy's law that the fluid velocity and pore pressure vary with the hydraulic permeability. Our study indicates that the poroelastic material property can affect the biomechanical responses to the mechanical stimulus and the permeability is important in controlling the fluid flow behaviours in the poroelastic model.

## 5. Conclusions

Although there have been some previous studies using FE analysis models mostly under compressive loads to examine biomechanical behaviours of the osteocyte-lacunar-canalicular system, these studies had not taken the bone poroelastic properties into account. The current study has developed a 3D poroelastic idealized FE model to investigate the biomechanical responses of the osteocyte-lacunar-canalicular system under different degrees of compressive loading stimuli. It was found that predicted maximum principal strains of osteocytes were ~633, 4272, 12820, and 21528; the maximum pore pressures were ~8.98, 74.24, 175.9, and 285.7 kPa; and the maximum fluid velocity values were ~2.69, 18.19, 56.65, and 97.98 *μ*m/s when the model was under 150 microstrains (disuse), 1000 microstrains (physiological), 3000 microstrains (overuse), and 5000 microstrains (pathological overload), respectively. The values of the strain, pore pressure, and fluid velocity, which were found to be the highest at the proximal region of cell processes, increase with the global loading. This new model can potentially be used to predict the mechanobiological behaviours of osteocytes under physiological or pathological loadings, which may provide an insight into understanding the mechanisms of mechanosensation and mechanotransduction of the bone.

## Figures and Tables

**Table 1:** Element numbers and element types of the finite element model of the osteocyte-lacunar-canalicular system.

Components	Number of elements	Element type
ECM	77480	Solid
PCM with canaliculi	7183	Solid
Osteocyte cell body and processes	23205	Solid

Total	107868	

ECM: extracellular matrix; PCM: perilacunar matrix.

**Table 2:** Properties of the poroelastic materials applied in the finite element model of the osteocyte-lacunar-canalicular system.

Components of osteocyte lacunae	Young's module	Poisson's ratio	Permeability *k*_0_ (m^2^)	Void ratio *e*_0_
ECM	11 GP [19]	0.38 [19]	1.0 × 10^−20^ [58]	0.053 [58]
PCM with canaliculi	40 kPa [59, 60]	0.4 [59, 60]	4 × 10^−20^ [59, 60]	4 [59, 60]
Osteocyte cell body and processes	3.1 kPa [45, 61]	0.35 [45, 61]	0.6 × 10^−19^ [45, 61]	4.88 [59, 60]

ECM: extracellular matrix; PCM: perilacunar matrix.

**Table 3:** Strain amplification factor comparisons between this study and previous studies.

Sources	Method	Strain amplification factor
Present method	FEM (poroelastic)	4.3
[24]	FEM (linearly isotropic)	3.14
[62, 63]	In vitro measurement	3.99–11.43
